# Fungal polysaccharides from *Inonotus obliquus* are agonists for Toll-like receptors and induce macrophage anti-cancer activity

**DOI:** 10.1038/s42003-024-05853-y

**Published:** 2024-02-23

**Authors:** Christian Winther Wold, Panagiotis F. Christopoulos, Maykel A. Arias, Deborah Elikplim Dzovor, Inger Øynebråten, Alexandre Corthay, Kari Tvete Inngjerdingen

**Affiliations:** 1 Section for Pharmaceutical Chemistry, Department of Pharmacy, University of Oslo, Oslo, Norway; 2 Tumor Immunology Lab, Department of Pathology, Rikshospitalet, Oslo University Hospital, Oslo, Norway; 3 Centro de Investigación Biomédica de Aragón (CIBA), University of Zaragoza, Zaragoza, Spain; 4 Hybrid Technology Hub – Centre of Excellence, Institute of Basic Medical Sciences, University of Oslo, Oslo, Norway; 5 Institute of Clinical Medicine, University of Oslo, Oslo, Norway

**Keywords:** Monocytes and macrophages, Tumour immunology

## Abstract

Fungal polysaccharides can exert immunomodulating activity by triggering pattern recognition receptors (PRRs) on innate immune cells such as macrophages. Here, we evaluate six polysaccharides isolated from the medicinal fungus *Inonotus obliquus* for their ability to activate mouse and human macrophages. We identify two water-soluble polysaccharides, AcF1 and AcF3, being able to trigger several critical antitumor functions of macrophages. AcF1 and AcF3 activate macrophages to secrete nitric oxide and the pro-inflammatory cytokines tumor necrosis factor-α (TNF-α) and interleukin-6 (IL-6). Combined with interferon-γ, the fungal polysaccharides trigger high production of IL-12p70, a central cytokine for antitumor immunity, and induce macrophage-mediated inhibition of cancer cell growth in vitro and in vivo. AcF1 and AcF3 are strong agonists of the PRRs Toll-like receptor 2 (TLR2) and TLR4, and weak agonists of Dectin-1. In comparison, two prototypical particulate β-glucans, one isolated from *I. obliquus* and one from *Saccharomyces cerevisiae* (zymosan), are agonists for Dectin-1 but not TLR2 or TLR4, and are unable to trigger anti-cancer functions of macrophages. We conclude that the water-soluble polysaccharides AcF1 and AcF3 from *I. obliquus* have a strong potential for cancer immunotherapy by triggering multiple PRRs and by inducing potent anti-cancer activity of macrophages.

## Abstract (web summary)

Two water soluble polysaccharides from the white rot fungus Inonotus obliquus mount a potent anti-tumour response by inducing anti-cancer activity of macrophages, hence can be strong candidates for cancer immunotherapy.

## Introduction

The efficacy of using immune cells to combat cancer in humans has been demonstrated by the success of immunotherapy based on immune checkpoint inhibitors that stimulate tumor-specific T cells^1,2^. Unfortunately, many cancer patients do not respond to current immunotherapies or develop secondary resistance, and novel treatment options are needed. Apart from T cells, other types of immune cells, such as macrophages, have a strong potential for cancer immunotherapy. Macrophages have a central role in cancer and can exert either anti- or pro-tumor activity in response to molecular cues from the tissue microenvironment^3–6^. Tumor-associated macrophages (TAMs) support tumor development by promoting angiogenesis and by dampening the antitumor immune response^4,7^. Accordingly, clinical trials have been launched with the aim of depleting or suppressing TAMs^5^. An alternative and promising strategy consists in activating TAMs so that they acquire an antitumor, so-called M1, phenotype^6,8^. M1-activated macrophages can exert a number of anti-cancer functions such as production of nitric oxide (NO) to kill cancer cells^9–11^, secretion of pro-inflammatory cytokines and chemokines to recruit immune cell into tumors^12,13^, inhibition of tumor angiogenesis^12^, and neoantigen presentation to T cells^3^.

The main function of macrophages in the immune system is to eliminate invading pathogens such as bacteria and fungi. To exert this task, macrophages possess an array of pattern recognition receptors (PRRs) that recognize evolutionary conserved molecules associated with different types of microbes^14^. Toll-like receptors (TLRs) are the best characterized family of PRRs^15^. Because most PRRs are activating receptors, they represent attractive targets to trigger the antitumor activity of macrophages. However, macrophages are multifunctional cells and the rules of macrophage activation remain to be fully elucidated. Accumulating evidence indicates that several macrophage receptors need to be triggered simultaneously to induce complete antitumor activity. For example, we have previously reported that a combination of a TLR agonist and an interferon (IFN-γ or IFN-α/β) is an efficient mean to induce antitumor M1 macrophages^9,10^. Yet, there is a strong medical need for novel compounds able to trigger robust antitumor activity of macrophages in vitro and in vivo for cancer immunotherapy.

Fungal polysaccharides, in particular β-glucans, have emerged as promising candidates for macrophage polarization into an M1 antitumor phenotype, due to their non-toxic nature and their potential to bind PRRs on macrophages^16–18^. Fungal β-glucans are polymers consisting of glucose in a β-anomeric configuration with (1→3) and/or (1→6) glycosidic linkages between the monomers. The C-type lectin Dectin-1, a PRR found on macrophages and dendritic cells, has been identified as a receptor for β-glucans from fungi and plants^19,20^. However, there is conflicting evidence regarding the immunostimulatory activity of fungal β-glucans, because β-glucan extracts tend not to be pure but rather a mix of various polysaccharides, proteins and lipoproteins. Several reports have shown that pure β-glucans do not induce the secretion of pro-inflammatory cytokines by macrophages and that the inflammatory activity of crude β-glucan extracts such as zymosan was due to contaminating TLR agonists^21,22^. Consequently, it remains unclear whether the binding of pure β-glucan to Dectin-1 does induce antitumor activity of macrophages.

*Inonotus obliquus*, a white-rot fungus found on birch trees and used in traditional medicine, is a promising source of bioactive compounds, and several authors have reported polysaccharides from *I. obliquus* with immunomodulatory and anti-cancer activity^23–26^. However, systematic characterization of the immunological properties of *I. obliquus* polysaccharides is lacking, and the immune receptors responsible for the activity remain to be identified. One study indicated that a crude polysaccharide extract from *I. obliquus* induced TLR2 signaling, but the extract was not chemically characterized^27^. We have previously isolated and purified polysaccharides from the water- and alkali extracts of the interior and exterior parts of *I. obliquus*. The polysaccharides were isolated after several purification steps in order to remove proteins and low molecular weight compounds, and were further fractionated using ion-exchange chromatography and size-exclusion chromatography, based on their charge or size, respectively^28^. We found that the water-extracted polysaccharides were complex and highly branched, and in addition to (1→3/1→6)-β-glucose (Glc), they contained several types of sugars, such as (1→6)-α-galactose (Gal), (1→4)-α-galacturonic acid (GalA), (1→3)-α-mannose (Man) and (1→4)-β-xylose (Xyl). The alkali-extracted polysaccharides had a simpler structural motif that resembled the classical β-glucans found in other fungi^28^.

Based on the ability to induce NO production by a macrophage cell line, six polysaccharides from *I. obliquus* were deemed promising for further testing and form the basis of the present study^28^. Here, we show that two of the water-extracted, acidic polysaccharide fractions, denominated AcF1 and AcF3, are agonists of TLR2, TLR4, and Dectin-1. Moreover, both AcF1 and AcF3 could synergize with IFN-γ to trigger production of NO and pro-inflammatory cytokines by macrophages and induce a macrophage phenotype with tumoricidal activity in vitro and in vivo. The ability of both AcF1 and AcF3 to activate multiple receptors on macrophages using one single molecule makes them attractive novel tools for cancer immunotherapy.

## Results

### Polysaccharides isolated from *I. obliquus* synergize with IFN-γ to induce NO production by macrophages

**Fig. 1:**
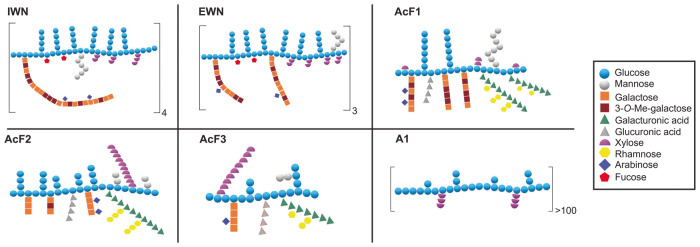
Tentative structures of the polysaccharides isolated from *I. obliquus* used in this study. The tentative structures of the polysaccharides are based on GC, GC-MS and NMR spectroscopic data^28^. The drawings are theoretical models, and do not necessarily reflect the three-dimensional shape of the polymers or the accuracy of the linkages between the different monomers. IWN and EWN are neutral polymers, with β-glucose and α-galactose being the main monomeric units. AcF1, AcF2, and, AcF3 are acidic polysaccharides with β-glucose as the main monomeric unit, and varying amounts of α-galacturonic acid, α-galactose and other monomers. A1 is an alkali-extracted polysaccharide with much higher molecular weight than the others, and consists almost exclusively of β-glucose, in addition to minor amounts of β-xylose, thus representing a prototypical β-glucan.

The main structural and chemical characteristics of the purified polysaccharides from *I. obliquus* that were used in this study are summarized in Table 1. The polysaccharides were allocated to three main types (Table 1), defined by their water-solubility (high or low), the absence (neutral) or presence (acidic) of GalA, and their molecular weight. Simplified structural models are shown in Fig. 1. The models represent probable polymer compositions based on gas chromatography - mass spectrometry (GC-MS) data and nuclear magnetic resonance (NMR) spectroscopy, and do not necessarily reflect the structural complexity or the accurate relationship between the monomers. Detailed chemical characterization of the polysaccharides has been previously reported^28^. While the water-soluble polysaccharides consisted of many types of monomers, the particulate and high molecular weight A1 polysaccharide consisted predominantly of (1→3)/(1→6)-β-Glc, thus representing a prototypical β-glucan.

**Table 1:** Structural characteristics of the polysaccharides isolated from *I. obliquus* used in this study, related to Fig. 1

*Inonotus obliquus* polysaccharide fractions	Abbreviation	Molecular weight (kDa)	Water solubility	Type	Main monomeric units
Interior part, water-soluble, neutral	IWN	60	High	Neutral	(1→3/1→6)-β-Glc (1→6)-α-Gal
Exterior part, water-soluble, neutral	EWN	73	High	Neutral	(1→3/1→6)-β-Glc (1→6)-α-Gal
Interior part, water-soluble, acidic fraction 1	AcF1	28	High	Acidic	(1→3/1→6)-β-Glc (1→6)-α-Gal (1→4)-α-GalA
Interior part, water-soluble, acidic fraction 2	AcF2	14	High	Acidic	(1→3/1→6)-β-Glc (1→6)-α-Gal (1→4)-α-GalA (1→4)-β-Xyl
Interior part, water-soluble, acidic fraction 3	AcF3	10	High	Acidic	(1→3/1→6)-β-Glc (1→6)-α-Gal (1→4)-α-GalA (1→4)-β-Xyl
Interior part, alkali-extracted, neutral	A1	>450	Low (particulate)	Neutral	(1→3/1→6)-β-Glc (1→4)-β-Xyl

Overview of names, abbreviations and the most important chemical characteristics of the polysaccharides used in this study, including molecular weight, water solubility, and main monomeric units. Neutral or acidic refers to the absence or presence of GalA, respectively.

**Fig. 2:**
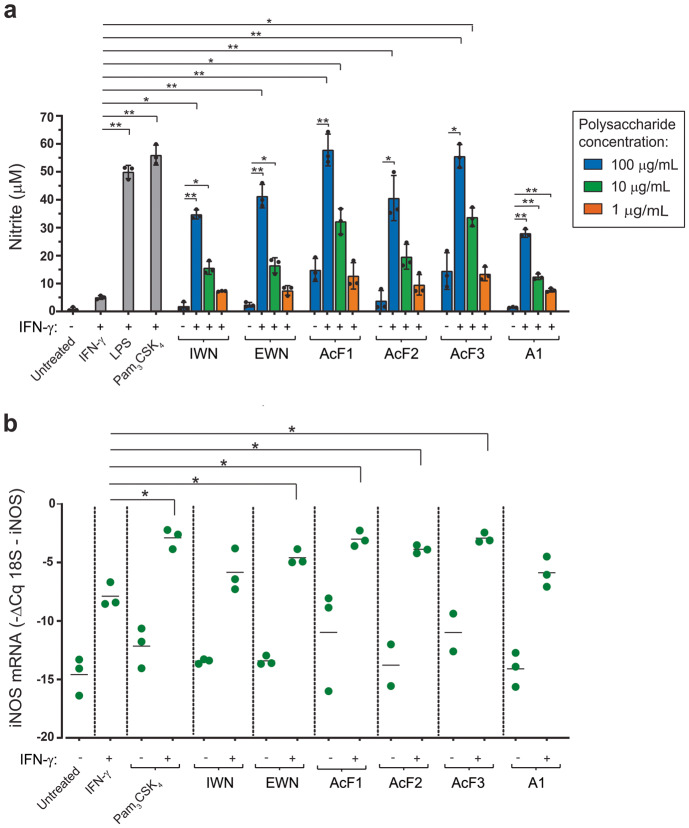
Polysaccharides from *I. obliquus* synergize with IFN-γ to induce NO production by mouse macrophages. **a** Bone marrow-derived macrophages (BMDMs) were incubated for 24 h with polysaccharides at different concentrations (1, 10 and 100 µg/mL) together with IFN-γ (20 ng/mL), or at 100 µg/mL alone, before nitrite levels in the supernatants were quantified using the Griess assay. Lipopolysaccharide (LPS) from *Escherichia coli* (100 ng/mL) and Pam_3_CSK_4_ (100 ng/mL) together with IFN-γ (20 ng/mL) were used as positive controls. Three independent experiments were performed, and the pooled data are shown as means ± SD. **b** BMDMs were incubated for 24 h with polysaccharides (100 µg/mL) with or without IFN-γ (20 ng/mL), before cells were harvested, mRNA was isolated and iNOS mRNA and 18 S rRNA were quantified using RT-qPCR. Pam_3_CSK_4_ (100 ng/mL) and IFN-γ (20 ng/mL) alone or in combination were used as controls. Each dot in the graph represents one experiment. The black lines indicate the means of two or three independent experiments. In (**a**) and (**b**), statistical significance was calculated using one-way ANOVA followed by Dunn’s multiple comparison test, with single comparisons against the IFN-γ-treated macrophages. In addition, in (**a**) statistical significance between + and – IFN-γ conditions for each individual sample concentration was calculated by a *t* test followed by Bonferroni correction. **p ≤ 0.01, *p ≤ 0.05.

The polysaccharides were first analyzed for their ability to induce NO production by macrophages, because NO is used by activated macrophages to kill cancer cells and NO production is an established marker for the M1 macrophage phenotype that is associated with anti-cancer activity^8,29^. Mouse bone marrow-derived macrophages (BMDMs) were used as a source of normal, non-immortalized macrophages, and incubated for 24 h with polysaccharides either alone or in combination with IFN-γ. The Griess assay was then used to measure NO in the form of nitrite in supernatants. Bacterial lipopolysaccharide (LPS) and synthetic triacylated lipopeptide Pam_3_CSK_4_ which bind to TLR4 and TLR1/2, respectively^30,31^, were used as positive controls. When used in combination with IFN-γ, all six polysaccharides from *I. obliquus* were, in a dose-dependent manner, capable of activating macrophages to secrete NO (Fig. 2a), which was in accordance with our previous observations with the J774 macrophage cell line^28^. For each of the five water-soluble polysaccharides, the NO levels were significantly higher when macrophages were treated with polysaccharides plus IFN-γ, in comparison with IFN-γ alone, which is consistent with a synergistic effect (Fig. 2a). Most of the polysaccharides needed to be combined with IFN-γ to induce NO. However, AcF1 and AcF3 were able to induce some NO secretion by macrophages in the absence of IFN-γ (Fig. 2a).

Next, we investigated the ability of the polysaccharides to induce the transcription of inducible nitric oxide synthase (iNOS), the enzyme that catalyzes the production of NO from L-arginine^32^. BMDMs were treated with polysaccharides in combination with or without IFN-γ, and iNOS mRNA levels were quantified (Fig. 2b). Compared to macrophages treated with IFN-γ only, the iNOS mRNA levels were significantly upregulated in macrophages treated with EWN, AcF1, AcF2, or AcF3, in combination with IFN-γ (Fig. 2b). The iNOS mRNA results were in accordance with the NO secretion levels (Fig. 2a), and confirm that the water-soluble polysaccharides (but not the particulate A1 β-glucan) synergize with IFN-γ to activate mouse macrophages to produce NO.

### Water-soluble polysaccharides from *I. obliquus* synergize with IFN-γ to induce tumoricidal activity of macrophages

**Fig. 3:**
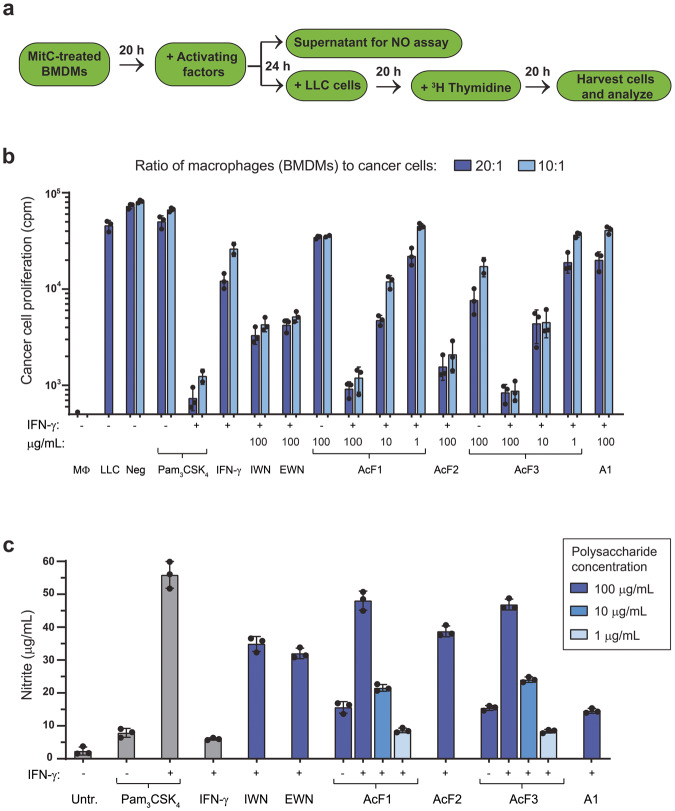
Polysaccharides from *I. obliquus* in combination with IFN-γ induce tumoricidal activity of macrophages. **a** Experimental setup: mouse macrophages (BMDMs, 6 × 10^4^ cells/well or 3 × 10^4^ cells/well for 20:1 and 10:1 ratios, respectively) were incubated with mitomycin C (MitC) for 2 h, and then cultivated for 20 h before treatment with polysaccharides ± IFN-γ for 24 h. Then, 100 µL cell medium was removed for NO quantification and Lewis lung carcinoma (LLC) cells (3 × 10^3^ cells/well) were added to the macrophages. After 20 h, radiolabeled thymidine was added and the co-cultures were incubated for another 24 h before cancer cell growth was measured by quantifying the incorporated thymidine. **b** Cancer cell growth was measured as radioactive counts per minute (cpm). **c** NO concentrations in the macrophage cell medium were measured using the Griess assay. The polysaccharide concentration was 100 µg/mL, except for AcF1 and AcF3, which were tested in concentrations 1, 10 and 100 µg/mL. All polysaccharides were tested alone and in combination with IFN-γ (20 ng/mL). Pam_3_CSK_4_ (100 ng/mL) and IFN-γ (20 ng/mL) were used as controls. Macrophages (MΦ) and LLC cells were also tested in a single culture. Untreated, negative control (Neg) consisted of co-cultured, non-activated BMDMs and LLC cells. Three independent experiments were performed, and a representative experiment is shown using average values ± SD from technical triplicates.

To evaluate the potential of the polysaccharides from *I. obliquus* to induce tumoricidal activity of macrophages, we used an in vitro growth inhibition assay that measures both cytotoxic and cytostatic activity of activated macrophages against cancer cells^9^. In this assay, BMDMs are first treated with mitomycin C to block their proliferation, before activation and subsequent co-culture with Lewis lung carcinoma (LLC) target cancer cells, whose growth is quantified by measuring the incorporation of radiolabeled thymidine (Fig. 3a). The growth of LLC cells was not affected by co-culture with non-activated BMDMs (‘Neg’ group in Fig. 3b). BMDMs activated with both Pam_3_CSK_4_ and IFN-γ were able to block almost completely the proliferation of LLC cells, as previously reported^9^. All polysaccharides, except the particulate A1 β-glucan, gave a dose-dependent inhibitory effect on cancer cell proliferation, with the most prominent effect seen for AcF1 and AcF3, in combination with IFN-γ (Fig. 3b). Inhibition of cancer cell growth was associated with elevated NO levels in the cell medium (Fig. 3c), which is consistent with cancer cell killing being mediated by NO secreted by activated macrophages^9–11^. Thus, water-soluble polysaccharides from *I. obliquus*, in particular AcF1, AcF2, and AcF3, are able to activate macrophages into a tumoricidal phenotype when combined with IFN-γ.

### AcF1, AcF2, and AcF3 activate TLR4-deficient macrophages to inhibit cancer cell growth

Contamination by bacterial LPS is a general concern when working with compounds of natural origin^33^. By use of GC-MS we have previously analyzed the purity of the polysaccharides isolated from *I. obliquus*, and no LPS contamination was detected in 100 µg/mL polysaccharide solutions. The detection limit of LPS in GC-MS was 1.4 ng/mL, as explained in detail in the Methods section^28^. To investigate further whether LPS was a contaminant, we performed an experiment using the LPS inhibitor polymyxin B (PMB), which neutralizes LPS by binding to its Lipid A moiety^34^. First, we confirmed that PMB could inhibit LPS-mediated NO secretion from macrophages by pre-incubating PMB with 100 ng/mL LPS from *Escherichia coli* (LPS-EK) or *Salmonella minnesota* (LPS-SM) (Supplementary Fig. 1a). Furthermore, in the presence of IFN-γ, NO production was nearly abolished by PMB at a LPS concentration of 10 ng/mL, and completely blocked at 1 ng/mL LPS (Supplementary Fig. 1a). In contrast, PMB pre-treatment did not influence the activity of the polysaccharides AcF1, AcF2, AcF3, or A1, whereas it seemed to slightly inhibit NO secretion induced by IWN and EWN (Supplementary Fig. 1b). Because PMB inhibited the activity of 10 ng/mL LPS, and 100 μg/mL polysaccharide solutions were previously shown to contain less than 1.4 ng/mL LPS (the GC-MS detection limit), any potential LPS contamination in our polysaccharide samples should be completely blocked by PMB. Therefore, we conclude that it is very unlikely that the observed activity of the polysaccharides AcF1, AcF2, AcF3, or A1 was due to LPS contamination.

**Fig. 4:**
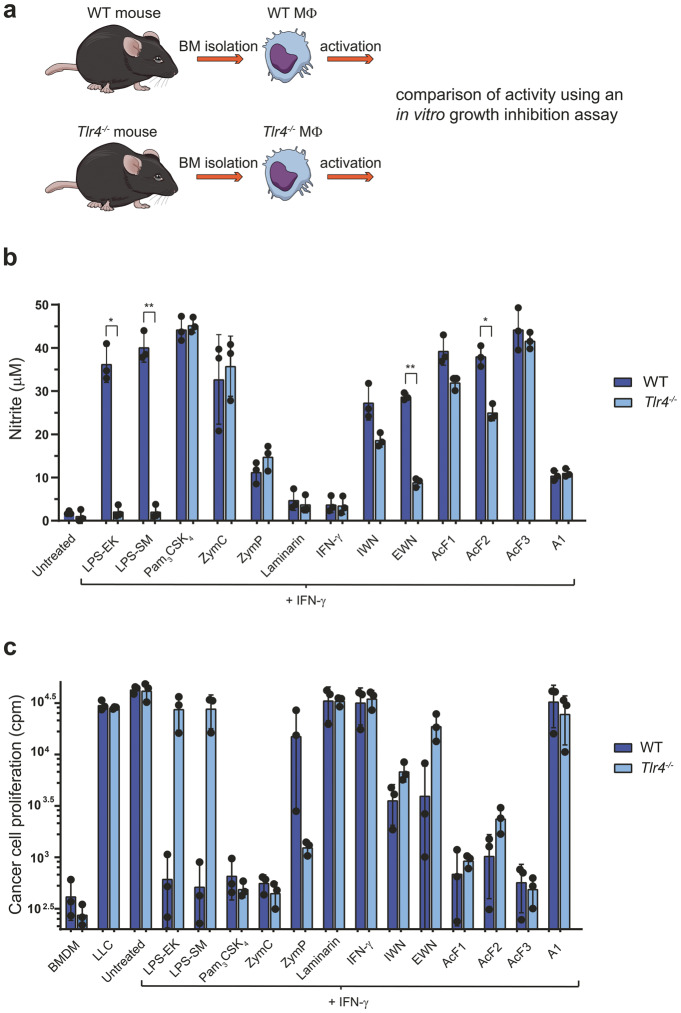
Polysaccharides from *I. obliquus* activate *Tlr4*^*−/−*^ macrophages to secrete NO and inhibit cancer cell growth. **a** Experimental design (created in the Mind the Graph platform; www.mindthegraph.com). BMDMs (MΦ) were generated from wild-type (WT) and *Tlr4*^*−/−*^ mice, and their activity was compared using an in vitro growth inhibition assay. BMDMs were incubated with mitomycin C for 2 h, before treatment with *I. obliquus* polysaccharides (100 µg/mL) and IFN-γ (20 ng/mL) for 24 h. LLC cells (3 × 10^3^/well) were added to stimulated BMDMs (3 × 10^4^ cells/well) at 10:1 ratio (BMDM:LLC). After 20 h, radiolabeled thymidine was added and the cells were incubated for 24 h before measurement of thymidine incorporation (cpm). LPS-EK, LPS-SM, Pam_3_CSK_4_ (all 100 ng/mL), zymosan crude (ZymC), zymosan purified (ZymP) and laminarin (all 100 µg/mL) together with IFN-γ were used as controls. **b** NO secreted into the cell medium of activated BMDMs was quantified using the Griess assay. **c** Growth inhibition of LLC cells by activated macrophages. Three independent experiments were performed, and average values ± SD are shown. Statistical significance indicates the difference between WT and *Tlr4*^*−/−*^ cells, and was calculated using a *t* test followed by Bonferroni correction. **p < 0.01, *p < 0.05.

To confirm that macrophage activation was mediated by the polysaccharides and not by LPS contamination, we used BMDMs generated from TLR4 knockout mice (*Tlr4*^*−/−*^), TLR4 being the receptor for LPS^30^. The activation of *Tlr4*^*−/−*^ and wild-type (WT) macrophages was compared after treatment with control ligands, or *I. obliquus* polysaccharides (100 µg/mL) in combination with IFN-γ (Fig. 4a). As expected, LPS-EK and LPS-SM were unable to activate *Tlr4*^*−/−*^ macrophages to produce NO. In contrast, the positive controls Pam_3_CSK_4_ (a TLR1/2 agonist) and zymosan crude (ZymC, a TLR2/Dectin-1 agonist) induced similar NO production by *Tlr4*^*−/−*^ and WT macrophages (Fig. 4b). All the polysaccharides from *I. obliquus* were able to induce NO production by *Tlr4*^*−/−*^ macrophages, i.e. in a TLR4-independent manner. For three polysaccharides, AcF1, AcF3, and A1, the NO levels were similar for WT and *Tlr4*^*−/−*^ macrophages. In contrast, IWN, EWN, and AcF2 showed significantly lower activity in cultures of *Tlr4*^*−/−*^ cells. Therefore, it cannot be excluded that LPS contamination contributes to the activity of IWN, EWN, and AcF2.

The tumoricidal activities of WT and *Tlr4*^*−/−*^ macrophages were compared in the growth inhibition assay. The three polysaccharides IWN, AcF1, and AcF3, activated WT and *Tlr4*^*−/−*^ macrophages to a similar extent, with AcF1 and AcF3 being the most potent (Fig. 4c). AcF2 also inhibited cancer cell growth, but showed a tendency of reduced activity with *Tlr4*^*−/−*^ macrophages. EWN was unable to induce tumoricidal activity of *Tlr4*^*−/−*^ macrophages, which suggests that the effect of EWN on macrophages was essentially mediated by TLR4 (and possibly LPS contamination). Intriguingly, zymosan purified (ZymP, a Dectin-1 agonist) was more potent at inducing tumoricidal activity of *Tlr4*^*−/−*^ macrophages as compared to WT macrophages, although the NO production was similar using the two cell types (Fig. 4c). Collectively, the data suggest that AcF1, AcF3, and A1 do not contain any LPS contamination, and demonstrate that AcF1, AcF2, and AcF3 are able to activate macrophages into a tumoricidal phenotype independently of TLR4.

### Polysaccharides from *I. obliquus* induce secretion of pro-inflammatory cytokines by mouse and human macrophages

**Fig. 5:**
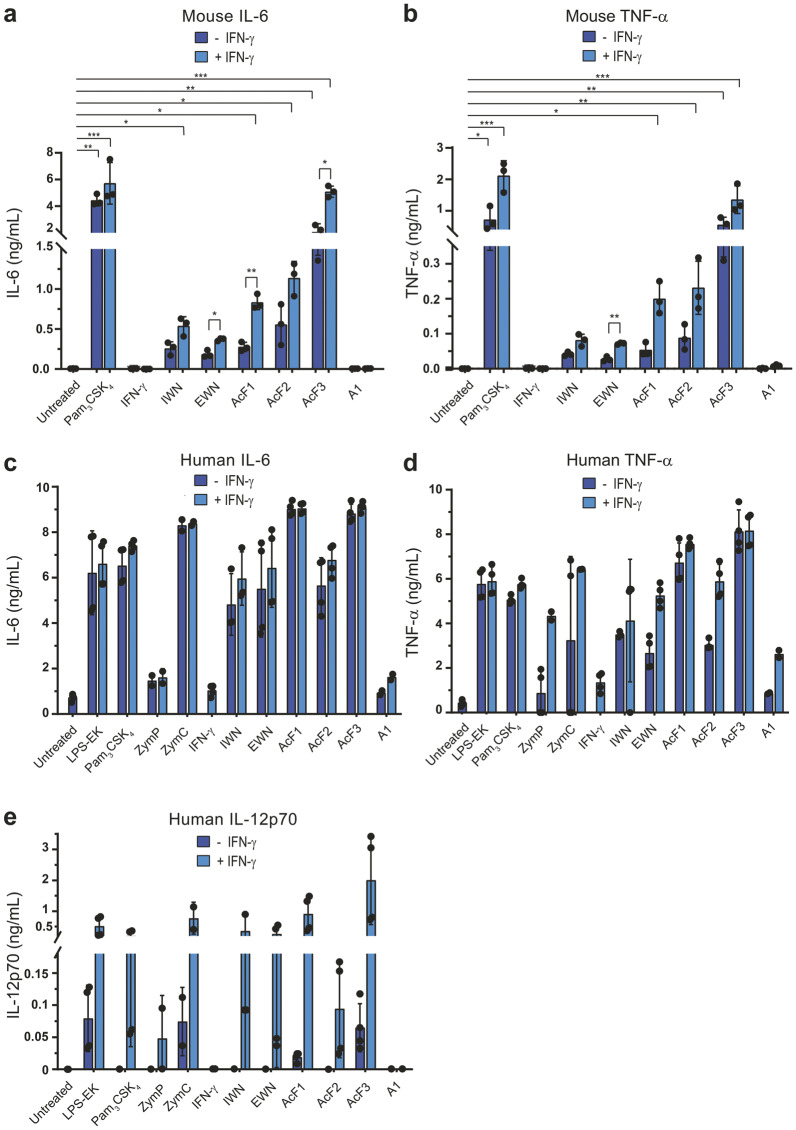
Polysaccharides from *I. obliquus* induce secretion of pro-inflammatory cytokines by mouse and human macrophages. Macrophages were treated with polysaccharides (100 µg/mL) alone or in combination with IFN-γ (20 ng/mL) for 24 h, and cytokine concentrations in supernatants were quantified by Luminex technology. **a**, **b** Production of IL-6 (**a**) and TNF-α (**b**) by mouse macrophages (2.5 × 10^5^ BMDMs/well). Pam_3_CSK_4_ (100 ng/mL) ± IFN-γ was used as a positive control. Three independent experiments were performed, and average values ± SD are shown. Statistical significance was calculated using one-way ANOVA followed by Dunn’s multiple comparison test, with single comparisons against the untreated control. In addition, statistical significance between + and – IFN-γ conditions for each individual sample concentration was calculated by a *t*-test followed by Bonferroni correction. ***p < 0.001, **p < 0.01, *p < 0.05. **c**–**e** Production of IL-6 (**c**), TNF-α (**d**), and IL12p70 (**e**) by human monocyte-derived macrophages (10^5^ cells/well) treated for 24 h with polysaccharides ± IFN-γ. LPS-EK, Pam_3_CSK_4_ (both 100 ng/mL), zymosan crude (ZymC, 100 µg/mL) and zymosan purified (ZymP, 100 µg/mL) were used as controls. Two independent experiments were performed, and a representative experiment is shown using average values ± SD.

In addition to direct killing of cancer cells (i.e. tumoricidal activity), activated M1 macrophages participate in tumor elimination by secreting pro-inflammatory cytokines that recruit other immune cells such as T cell into tumors^12,35^. To test whether polysaccharides from *I. obliquus* could induce cytokine secretion by macrophages, mouse BMDMs and human monocyte-derived macrophages were stimulated for 24 h with *I. obliquus* polysaccharides, with and without IFN-γ, before cytokines were quantitated in the cell culture medium. Whereas the particulate A1 β-glucan was ineffective, all the water-soluble polysaccharides could activate mouse macrophages to secrete the pro-inflammatory cytokines interleukin (IL)−6 and TNF-α (Fig. 5a, b). Combined treatment with IFN-γ resulted in higher cytokine levels, in particular for AcF1, AcF2, and AcF3. AcF3 showed the strongest effect and was also able to induce significant levels of both IL-6 and TNF-α in the absence of IFN-γ (Fig. 5a, b). All polysaccharides from *I. obliquus* were able to induce secretion of IL-6 and TNF-α by human macrophages, although A1 had much lower activity than the others (Fig. 5c, d). Notably, the water-soluble polysaccharides induced IL-6 and TNF-α secretion by human macrophages at levels comparable to the positive controls LPS-EK and Pam_3_CSK_4_. The addition of IFN-γ showed minor or no additional effect on the secretion of IL-6 and TNF-α by human macrophages (Fig. 5c, d). Finally, we measured IL-12p70 secretion by human macrophages, because IL-12p70 is a crucial cytokine for the induction of type 1 immunity that protects against cancer^12,36^. All the water-soluble polysaccharides were found to synergize with IFN-γ to induce the production of copious amounts of IL-12p70 by human macrophages, at levels similar to the positive controls LPS and Pam_3_CSK_4_ (Fig. 5e). AcF3 was also able to induce low levels of IL-12p70 in the absence of IFN-γ, which is remarkable. Interestingly, when compared to Pam_3_CSK_4_, all the polysaccharides from *I. obliquus* seemed to be more active on human than on mouse macrophages. In summary, the results demonstrate the pro-inflammatory activity of the polysaccharides from *I. obliquus*, with AcF3 being the most potent fraction, activating macrophages to secrete high levels of IL-6, TNF-α, and IL-12p70.

### The *I. obliquus* polysaccharides trigger human TLR2, TLR4, and Dectin-1a

**Fig. 6:**
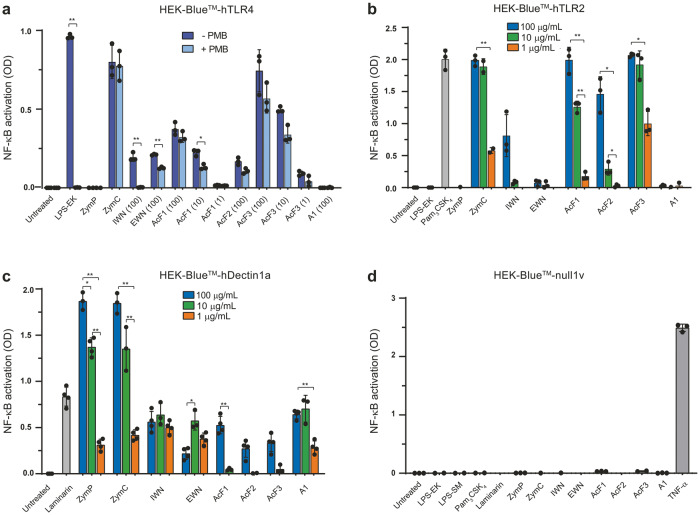
*I. obliquus* polysaccharides are agonists for the human immune receptors TLR4, TLR2, and Dectin-1a. **a**–**c** HEK-Blue™ cells transfected with human TLR4 (**a**), TLR2 (**b**), or Dectin1a (**c**) were incubated with polysaccharides or controls for 16 h. OD_635_ was measured in order to detect Secreted Embryonic Alkaline Phosphatase (SEAP) in the supernatants. Polysaccharides were used at 1, 10, and 100 µg/mL. **d** For the null cells, polysaccharides were used at concentration 100 µg/mL. LPS-EK (10 ng/mL), Pam_3_CSK_4_ (100 ng/mL), laminarin (100 µg/mL), zymosan crude (ZymC) and zymosan purified (ZymP) (both at 1, 10, and 100 µg/mL) and TNF-α (10 ng/mL) were used as controls depending on the reporter cell line. PMB concentration was 10 µg/mL in (**a**). Three (**a**, **b**, **d**) or four (**c**) independent experiments were performed, and average values ± SD are shown. In **(a)** statistical significance between + and – PMB conditions for each individual sample concentration was calculated by a *t* test followed by Bonferroni correction, and in (**b**) and (**c**) statistical significance between each ligand dose was calculated by a *t* test followed by Bonferroni correction. **p < 0.01, *p < 0.05.

To identify the macrophage receptors that are triggered by the fungal polysaccharides, we used reporter HEK-Blue™ cell lines transfected with specific human PRRs. In this assay, interaction between a ligand and the transfected receptor causes activation of the transcription factors NF-κB and AP-1 which in turn induce the production of secreted embryonic alkaline phosphatase (SEAP). The levels of SEAP are quantified in cell culture medium. First, we investigated whether *I. obliquus* polysaccharides could stimulate human TLR4 (hTLR4). All samples, polysaccharides and controls, were either pre-treated with the LPS inhibitor PMB or left untreated. As anticipated, the positive control LPS-EK could activate HEK-hTLR4 whereas PMB-pre-treated LPS-EK gave no detectable signal (Fig. 6a). Among the *I. obliquus* polysaccharides, AcF1 and AcF3 were found to be the most potent TLR4 agonists, and the activity was retained after treatment with PMB (Fig. 6a). This was an unexpected finding because the activity of AcF1 and AcF3 was similar toward WT and *Tlr4*^*−/−*^ macrophages in the growth inhibition assay (Fig. 4), which suggested that AcF1 and AcF3 did not activate TLR4. Thus, it appears that AcF1 and AcF3 possess both TLR4-dependent and TLR4-independent immunomodulatory activity. EWN and AcF2 were also able to activate TLR4, although to a lower degree, and, according to the *Tlr4*^*−/−*^ experiments, it could not be ruled out that this activation was due to LPS contamination.

Next, we used HEK reporter cells transfected with human TLR2, since it has been reported that fungal and plant polysaccharides are able to activate macrophages through this receptor^37,38^. AcF1 and AcF3 were found to strongly activate hTLR2, at levels comparable to the TLR1/2 agonist Pam_3_CSK_4_ (Fig. 6b). EWN and A1 were inactive, whereas AcF2 and IWN were only active at the highest concentration tested (100 µg/mL). A crude formulation of zymosan (ZymC) containing various compounds including mannans and lipoproteins showed potent activity against TLR2. Notably, zymosan lost its TLR2-triggering capability when used as the purified “β-glucan only” formulation (ZymP), as previously reported^22,39^.

Finally, we tested whether the *I. obliquus* polysaccharides could activate human Dectin-1a, a known β-glucan receptor^19,40^. All six polysaccharides activated the hDectin-1a receptor, but at varying degrees (Fig. 6c). The activity of the polysaccharides from *I. obliquus* was lower than that mediated by the particulate β-glucan zymosan (both purified and crude) but had comparable activity to the water-soluble β-glucan laminarin. Interestingly, IWN, EWN, and A1, which had only shown limited activity in the NO, growth inhibition, and cytokine assays, were the most potent hDectin-1a agonists. Even when used at a relatively low concentration of 1 µg/mL, IWN, EWN, or A1, were able to trigger signaling via Dectin-1a (Fig. 6c). The acidic polysaccharides AcF1, AcF2, and AcF3 gave detectable hDectin-1a activation, but only at the highest concentration tested (100 µg/mL). None of the polysaccharides could activate the control HEK-Blue™ null cells, confirming that the results obtained were due to the specified transfected receptors (Fig. 6d). Taken together, the data show that AcF1 and AcF3 are the most active PRR agonists of the isolated *I. obliquus* polysaccharides; AcF1 and AcF3 were able to activate both TLR2 and TLR4 and to some extent Dectin-1a, although tumoricidal macrophage activation in vitro did not require activation of TLR4.

### Macrophages activated by AcF1 or AcF3 prevent tumor development in vivo

**Fig. 7:**
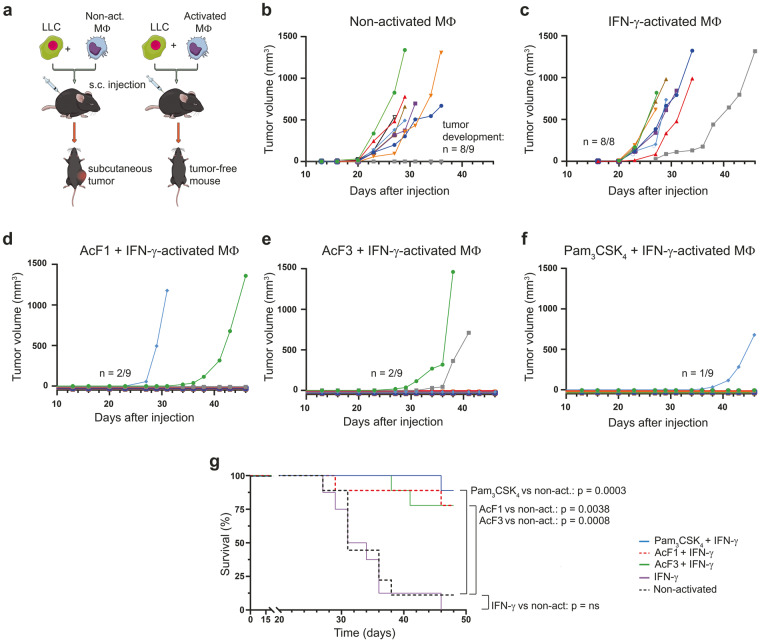
Macrophages activated by AcF1 or AcF3 in combination with IFN-γ prevent tumor development in vivo. BMDMs (MΦ) were treated in vitro with AcF1 or AcF3 polysaccharides from *I. obliquus* (100 µg/mL), in combination with IFN-γ (20 ng/mL), for 24 h to induce an antitumor M1 macrophage phenotype. Macrophages treated with Pam_3_CSK_4_ (100 ng/mL) in combination with IFN-γ, IFN-γ alone, and untreated macrophages were used as controls. Next, the macrophages were mixed with cancer cells at a ratio of 15:1 (7.5 ×105 BMDMs and 5 ×104 LLC cells per mouse) and injected subcutaneously into syngeneic C57BL/6 mice (*n* = 9 per group). Tumor growth was monitored over time. **a** Experimental design (created in the Mind the Graph platform; www.mindthegraph.com). **b**–**f** Growth curves showing the tumor volume (mm^3^) throughout the experimental period. Each line in the graphs indicates one individual mouse. **g** Kaplan–Meier plot showing survival curves for each group of mice. *P* values were calculated by comparing each group with the non-activated macrophage control group.

The data presented in Fig. 4 revealed that polysaccharides from *I. obliquus*, in combination with IFN-γ, could induce tumoricidal activity of macrophages in vitro, resulting in potent inhibition of cancer cell growth. To test the in vivo relevance of these observations, we performed an experiment in which macrophages were first activated in vitro, and then mixed with LLC cancer cells immediately before subcutaneous co-injection into syngeneic mice (Fig. 7a). AcF1 and AcF3, the two polysaccharides from *I. obliquus* with the strongest immunostimulatory capacity, were selected for this experiment which included five groups: non-activated BMDMs, and BMDMs activated with AcF1 + IFN-γ, AcF3 + IFN-γ, IFN-γ alone, or Pam_3_CSK_4_ + IFN-γ (positive control). Tumor development was recorded over time after the co-injections. Among the mice that received non-activated macrophages or IFN-γ-alone-treated macrophages, all animals except one developed tumors (Fig. 7b, c). In sharp contrast, most of the mice injected with macrophages activated with AcF1 + IFN-γ, AcF3 + IFN-γ, or Pam_3_CSK_4_ + IFN-γ, remained tumor-free for the duration of the experiment (Fig. 7d–f). In the two groups with macrophages activated with polysaccharide plus IFN-γ, only two out of nine mice developed tumors, forty-eight days after the co-injections (Fig. 7d, e). Statistical analysis showed that co-activation of macrophages with AcF1 + IFN-γ or AcF3 + IFN-γ significantly increased the survival of the mice compared to the non-activated or IFN-γ-only-activated groups (Fig. 7g). These co-injection experiments demonstrate the ability of AcF1 and AcF3, when combined with IFN-γ, to induce potent tumoricidal activity of macrophages, leading to in vivo elimination of LLC cancer cells and thereby prevention of tumor development in mice.

## Discussion

In this paper, we investigated the ability of six polysaccharides isolated from the medicinal fungus *I. obliquus* to activate macrophages into a tumoricidal phenotype. The results are summarized in Table 2. Based on their structural motifs and molecular weight, the polysaccharides were allocated to three main structural types (Table 1), defined as neutral and water-soluble (IWN and EWN), acidic and water-soluble (AcF1, AcF2, and AcF3), or neutral and particulate (A1). The acidic polysaccharides appeared to be more potent at activating macrophages, in combination with IFN-γ, compared to the neutral ones. In particular, AcF1 and AcF3 demonstrated a strong inhibition of cancer cell growth both in vitro and in vivo, through activation of macrophages. The in vivo growth inhibition assay showed complete prevention of tumor development in most mice given macrophages activated with AcF1 or AcF3 in combination with IFN-γ. Collectively, the data indicate a strong potential of AcF1 and AcF3 for cancer immunotherapy, by demonstrating the capacity of AcF1 and AcF3 to induce a complete antitumor M1 macrophage phenotype, as defined by NO production, induction of pro-inflammatory cytokines, and inhibition of cancer cell growth.

**Table 2:** Macrophage activation and receptor triggering by *I. obliquus* polysaccharides

		Macrophage activation					Receptor triggering			LPS Contami-nation^a^	Potential for immuno-therapy^b^
	Origin	NO production	Inhibition of cancer cell growth	Mouse IL-6, TNF-α	Human IL-6, TNF-α	Human IL-12p70	Dectin-1a	TLR2	TLR4		
**IWN**	*I. obliquus*	++	+	+	++	++	++	+	(+)	(+)	Low
**EWN**	*I. obliquus*	++	+	+	++	++	++	-	(+)	(+)	Low
**AcF1**	*I. obliquus*	+++	+++^c^	++	+++	+++	+	+++	++	−	High
**AcF2**	*I. obliquus*	++	++	++	++	+	+	++	+	(+)	Medium
**AcF3**	*I. obliquus*	+++	+++^c^	+++	+++	+++	+	+++	+++	−	High
**A1**	*I. obliquus*	+	(+)	−	(+)	−	++	−	−	−	Low
**LPS-EK**	*E. coli*	+++	+++	n.t.	+++	+++	n.t.	−	+++	n.r.	n.r.
**Pam**_**3**_**CSK**_**4**_	synthetic	+++	+++^c^	+++	+++	++	n.t.	+++	n.t.	-	n.r.
**ZymC**	*S. cerevisiae*	+++	+++	n.t.	+++	+++	+++	+++	+++	+	n.r.
**ZymP**	*S. cerevisiae*	+	+	n.t.	(+)	+	+++	−	−	−	n.r.
**Laminarin**	*L. digitata*	−	−	n.t.	n.t.	n.t.	++	n.t.	n.t.	−	n.r.

The table is based on a summary of the experiments carried out in this study, giving each *I. obliquus* polysaccharide a grade ranging from – to +++ depending on its potency to activate macrophages in synergy with IFN-γ in the indicated assay, or to trigger receptor signaling. (+), very low activity; +, low; ++, intermediate; +++, high; -, none; n.t., not tested; n.r., not relevant.

^a^Grading of possible LPS contamination is based on a comparison between activities when the polysaccharides were pre-treated or not with the LPS antagonist polymyxin B.

^b^The potential for immunotherapy was evaluated based on the ability to induce the anti-cancer activities of macrophages in vitro. For AcF1 and AcF3, the ability to prevent tumor development in vivo in mice was also tested.

^c^Tested both in vitro and in vivo.

The capability of the acidic polysaccharides (AcF1, AcF2, and AcF3) at inducing antitumor M1 macrophages seemed to correlate with the ability to activate TLR2 and/or TLR4 (Table 2). The main difference between the acidic and neutral polysaccharides was the presence of 12-17% (1→4)-α-galacturonic acid (GalA) in the acidic polysaccharides, suggesting that GalA might be crucial for the activity of AcF1 and AcF3. Citrus pectin containing mainly (1→4)-α-GalA has previously been reported to activate both TLR2 and TLR4, and the activation of TLR2 was dependent on a high degree of methyl esterification of the GalA units^41^. These findings suggest that GalA is important for triggering TLR2 and TLR4. Activation of TLR2 may possibly require a combination of GalA with other structural motifs present in the acidic polysaccharides, such as β-glucose and mannose-containing motifs^28,42^. An acetylated glucomannan polysaccharide isolated from konjac root has been reported to induce macrophages with an antitumor phenotype through activation of TLR2^42^. Further, it might be that the observed differences in activity of the polysaccharides are caused by variations in their three-dimensional structure, which could explain why AcF2 was less active than AcF1 and AcF3. According to GC-MS data^28^, AcF2 exhibited a higher degree of neutral side-chains than AcF1 and AcF3, which could affect the accessibility of the GalA towards TLR2 and TLR4.

AcF1 and AcF3 were able to induce NO production by mouse macrophages without co-treatment with IFN-γ (Fig. 2), a finding which might be explained by the ability of AcF1 and AcF3 to activate TLR4. We have previously reported that the combination of a TLR agonist with either a type I interferon (IFN-α/β) or with the type II interferon IFN-γ was an efficient means to induce antitumor M1 macrophages^9,10^. Strikingly, macrophage activation by TLR agonists in the absence of interferon was inefficient with the exception of high dose of the TLR4 agonist LPS. Notably, TLR4 is a unique TLR that is linked to both the MyD88 and the TRIF signaling pathways. TLR4-mediated activation of the TRIF signaling pathway results in autocrine production of the type I interferons IFN-α/β by macrophages. The ability of high dose LPS to induce tumoricidal activity of macrophages in the absence of IFN-γ was shown to depend on induction of autocrine type I IFNs^10^. Therefore, it is tempting to speculate that the ability of AcF1 and AcF3 to induce NO production by macrophages in the absence of IFN-γ may similarly be due to the induction of type I IFNs via stimulation of TLR4.

In addition to TLR2 and TLR4, some of the *I. obliquus* polysaccharides were able to activate human Dectin-1a. Dectin-1 is a well-characterized β-glucan receptor on macrophages, recognizing fungal β-glucans with high affinity^19,20^. Because all the *I. obliquus* polysaccharides had a main structural motif resembling fungal β-glucans, their ability to activate Dectin-1 was not surprising. However, the acidic polysaccharides AcF1, AcF2 and AcF3 showed weak activation of Dectin-1a compared to the neutral fractions IWN, EWN and A1. Affinity for Dectin-1 is known to vary depending on the conformation of the β-glucan backbone^43^. It is likely that the β-glucan parts of IWN, EWN and A1 were more exposed to the outer environment compared to AcF1, AcF2 and AcF3, resulting in higher affinity for Dectin-1a. Notably, the Dectin-1a activity was relatively weak for all *I. obliquus* polysaccharides compared to the particulate β-glucan zymosan, but was comparable to the soluble β-glucan laminarin (Table 2).

Dectin-1 is a PRR that enables macrophages to recognize β-glucan-bearing microbes such as fungi and bacteria. Binding of β-glucan to Dectin-1 triggers cellular antimicrobial activity, including phagocytosis and production of reactive oxygen species (ROS) in phagosomes to eliminate the internalized pathogen^21,44,45^. Dectin-1 signaling requires clustering of two Dectin-1 molecules^45,46^. Unlike other PRRs such as TLRs, Dectin-1 discriminates between soluble and particulate ligands. Dectin-1 binds to both soluble and particulate β-glucans, but ROS production in mouse and human macrophages was shown to be induced only by particulate β-glucans^40^. Particulate β-glucans were shown to cluster the Dectin-1 receptors in synapse-like structure from which regulatory tyrosine phosphatases (CD45 and CD148) were excluded^40^. It has been proposed that this mechanism allows macrophages to distinguish direct microbial contact from detection of microbes at a distance, thereby initiating cellular antimicrobial responses (phagocytosis and ROS production) only when required^40^. Beyond the induction of phagocytosis and ROS, the inflammatory activity of β-glucans has been questioned, because β-glucan extracts tend not to be pure but rather a mix of various polysaccharides, proteins and lipoproteins^21,22^. The reported ability of β-glucans to induce secretion of pro-inflammatory cytokines by macrophages seemed to be dependent on contaminating TLR agonists^21,22,47^. In accordance with this view, the two pure particulate β-glucans that we tested, namely the A1 polysaccharide from *I. obliquus* and zymosan depleted from *S. cerevisiae*, were both poor inducers of IL-6 and TNF-α secretion by macrophages (Table 2). In fact, the data presented in Table 2 strongly suggest that pure particulate β-glucans are non-inflammatory and unable to induce antitumor M1 macrophages.

The two most potent polysaccharides from *I. obliquus*, AcF1 and AcF3, were found to be strong agonists of TLR2 and TLR4 and weak agonists for Dectin-1 (Table 2). The capacity to trigger several PRRs is a remarkable feature of the *I. obliquus* polysaccharides because it could potentially lead to synergistic activation of macrophages. In fact, several reports have shown that Dectin-1 may collaborate with TLR2 or TLR4 for macrophage activation. For example, in response to crude zymosan, Dectin-1 and TLR2 were reported to synergize for NF-κB activation^21^, and the production of TNF-α by BMDMs infected with mycobacteria was shown to be dependent on Dectin-1 and TLR2 acting in synergy^48^. Other studies reported that Dectin-1 synergized with both TLR2 and TLR4 for TNF-α production by human monocyte-derived macrophages and dendritic cells^49,50^. The synergistic immune signaling mediated by Dectin-1 in combination with TLR2 requires a proximity <500 nm between the two PRRs^51^. Pure particulate β-glucan has been shown to synergize with the TLR2/1 agonist Pam_3_CSK_4_ for TNF-α production by mouse macrophages, and the synergy seemed to depend on both the Syk signaling pathway that is downstream of Dectin-1 and the MyD88 signaling pathway downstream of TLR2. Collaboration of the Syk and TLR/MyD88 pathways was shown to result in sustained degradation of the inhibitor of kB (IkB), thereby enhancing NF-κB nuclear translocation^52^. Thus, collaboration between PRRs for macrophage activation is likely to depend on signal integration by connected signaling pathways.

TLR agonists are considered promising new treatments for cancer and three TLR agonists have already been approved by regulatory agencies^53^. Considering clinical use of polysaccharides, lentinan is a β-glucan isolated from *Lentinus edodes* (shiitake), exhibiting antitumor effects by affecting T helper type 1 cells and macrophages. ^54^ Parenteral injections of lentinan are applied as adjuvant treatment in gastric cancer and colorectal cancer. ^55,56^ In addition to the possibility of parenteral administration, a lymphatic route via the gut wall barrier has been described for polysaccharides. A hyperbranched heteroglycan isolated from *Astragalus membranaceus* was recently reported to be absorbed into the lymphatic system through M cells and Peyer’s patches. ^57^ M cells are specialized epithelial cells, delivering pathogens and food molecules from the gut lumen to the lymphatic system. ^58^

In conclusion, we here report the immunological activity of several polysaccharides isolated from *I. obliquus*, with the acidic, water-soluble polysaccharides AcF1 and AcF3 being able to induce a potent tumoricidal macrophage phenotype both in vitro and in vivo. The high water-solubility of AcF1 and AcF3 will be an advantage for in vivo delivery compared to particulate β-glucan formulations. The ability of AcF1 and AcF3 to activate multiple receptors on macrophages using one single molecule and to induce a potent antitumor M1 macrophage phenotype makes them attractive novel tools for cancer immunotherapy.

## Methods

### Isolation and characterization of polysaccharides from *I. obliquus*

Sclerotia (dense fungal conks) of *I. obliquus* were harvested from a birch (*Betula pubescens*) tree growing in Oslo, Norway, and polysaccharides were isolated and characterized as previously described^28^. Briefly, dried material from the interior part of *I. obliquus* (IOI) or the exterior part of *I. obliquus* (IOE) was boiled in water or 1 M NaOH, before the extracts were precipitated using 70% EtOH and dialyzed (cut-off 3.5 kDa) in distilled H_2_O. Column chromatography was used to fractionate the extracts. The water-soluble extracts were first applied to an ion-exchange column, and the neutral fractions (N) were eluted with distilled H_2_O whereas a linear NaCl gradient (0–1.5 M) was used to obtain the acidic fractions (Ac). Two neutral, water-soluble extracts were isolated, one from the interior part (IWN) and one from the exterior part (EWN). The acidic fractions (Ac) were obtained from the interior part, and after ion-exchange chromatography, they were separated further using a size-exclusion column to yield AcF1, AcF2, and AcF3. The alkali extract from the interior part was applied to a size-exclusion column to yield the neutral A1 polysaccharide. The fractions were characterized using several different methods, including gas chromatography (monosaccharide composition), GC-MS (linkage analysis), 2D NMR spectroscopy (anomeric configuration), size exclusion chromatography – multiple-angle laser light scattering (SEC-MALLS, for molecular weights) and Smith degradation (relationship between parts of the polymer)^28^. For simplicity, the polysaccharide names were shortened here as compared to our previous publication:^28^ IOI-WN → IWN; IOI-WAcF1 → AcF1; IOI-WAcF2 → AcF2; IOI-WAcF3 → AcF3; IOE-WN → EWN; IOI-A1 → A1.

### Mice

C57BL/6NRj mice were purchased from Janvier Labs (Le Genest-Saint-Isle, France) and bred at the Department of Comparative Medicine, Oslo University Hospital, Rikshospitalet, Oslo, Norway, in specific pathogen free (SPF) conditions. C57BL/6NRj mice deficient in TLR4 (*Tlr4*^*−/−*^)^30^ were bred at the University of Zaragoza, Zaragoza, Spain. The study was approved by the Norwegian Food Safety Authority (approval number 20/102031), and all the experiments were performed in accordance with the national regulations and the EU directive 2010/63/EU.

### Isolation and differentiation of mouse bone marrow-derived macrophages

Conditioned medium containing macrophage colony-stimulating factor (M-CSF) was produced by culturing L929 cells in RPMI 1640 medium (Thermo Fisher Scientific) containing 10% fetal bovine serum (FBS, Biochrom GmbH). After 10 days, the conditioned medium was collected, centrifuged, passed through a 0.22 μm filter, and stored at −20 °C until use for the differentiation and maintenance of bone marrow-derived macrophages (BMDMs). For isolation of bone marrow cells, femurs and tibiae of the hind legs from 8-to-12-week-old, male and female C57BL/6NRj mice and 8-week-old, female C57BL/10ScN *Tlr4*^*−/−*^ knockout mice were harvested and flushed with RPMI 1640 medium containing 10% FBS under sterile conditions. Bone marrow cells that passed through a cell strainer with 70 μm pores (Sigma-Aldrich) were cultured in non-tissue culture treated dishes (10 cm, VWR) in RPMI 1640 medium containing 10% FBS, and 30% L929-derived conditioned medium. The cells were cultured for 5 days, after which non-adherent cells were washed off using phosphate-buffered saline (PBS, with MgCl_2_ and CaCl_2_, Sigma-Aldrich) and the adherent macrophages were cultured for 2 more days. Macrophages were then harvested by incubation (20 min at 4 °C) with cold PBS (without CaCl_2_ and MgCl_2_, Sigma-Aldrich). Next, the macrophages were flushed off the plate, collected, counted and kept frozen in aliquots at −150 °C in FBS with 10% dimethyl sulfoxide (DMSO, VWR) for future experiments. The purity of the cells was 99% as analyzed by flow cytometry using the macrophage markers CD11b (clone M1/70, BioLegend) and F4/80 (clone BM8, BioLegend).

### Isolation and differentiation of human monocyte-derived macrophages

Buffy coats were obtained from the Blood bank of Oslo University Hospital and approved for use by the Norwegian Regional Committee for Medical and Health Research Ethics (REK no. 2019/113). All ethical regulations relevant to human research participants were followed. Buffy coat mixed with an equal volume of PBS containing 2% FBS, was gently added to Lymphoprep™ (Progen) in 50 mL tubes in volumes recommended by the provider. After centrifugation at 800 g for 20 min at room temperature, the middle, buffy layer containing peripheral blood mononuclear cells was collected and washed twice in PBS by centrifugation (400 g, 7 min, room temperature). Next, the cells were filtered through a 30 µm filter to remove cell clumps and debris, before the monocytes were positively selected by magnetic-activated cell sorting (MACS) technology (Miltenyi) according to the manufacturer’s instructions. Briefly, magnetic beads conjugated to an anti-human CD14 antibody (CD14 MicroBeads, Miltenyi) were added to the mononuclear cells and incubated for 15 min at 4 °C. Next, the cell suspension was applied onto a magnetic column (Miltenyi). Unlabeled cells were washed away before the labeled cells were flushed out. Staining of the eluted cells with APC/Cy7-conjugated anti-human CD14 antibody (clone HCD14, BioLegend) followed by flow cytometry, showed that >95% of the positively selected cells were monocytes. APC/Cy7-conjugated mouse IgG1k (clone MOPC-21, BioLegend) was used as an isotype-matched control antibody.

The positively selected CD14^+^ monocytes were differentiated into macrophages by cultivation for 6 days in medium with macrophage colony-stimulating factor (M-CSF, Peprotech). More specifically, 3 × 10^6^ cells were seeded out in 10 mL RPMI 1640 containing 10% FBS and 50 ng/mL M-CSF per 10 cm non-tissue culture treated dish (VWR). At day 3, half of the medium was replenished with fresh medium containing 50 ng/mL M-CSF. On day 6, macrophages were harvested by first collecting culture medium into polypropylene falcon tubes (50 mL, SARSTEDT) pre-coated with FBS. The tubes were kept on ice. Then, 10 mL of detachment buffer made in-house, consisting of PBS (without CaCl_2_ and MgCl_2_), 2.5 mM EDTA, and 1% FBS, was added to each culture plate and incubated at 37 °C for 30 min. Next, dislodged cells were collected by pipetting up and down several times. A cell scraper was used to detach the remaining macrophages.

### PRR agonists and cytokines

The following PRR agonists were used as controls in various experiments: synthetic triacylated lipopeptide CysSerLys_4_ (Pam_3_CSK_4_, a TLR1/TLR2 agonist, InvivoGen); Lipopolysaccharide from *Escherichia coli* K12 strain (LPS-EK) and *Salmonella minnesota* (LPS-EM) (both ultrapure TLR4 agonists, InvivoGen); Lipoteichoic acid (LTA) from *Staphylococcus aureus* (TLR2/TLR6 agonist, Sigma-Aldrich); CL264 (TLR7 agonist, InvivoGen); zymosan from *Saccharomyces cerevisiae* (zymosan crude/ZymC, TLR2 and Dectin-1 agonist, Sigma-Aldrich); zymosan depleted from *S. cerevisiae* (zymosan purified/ZymP, InvivoGen); and laminarin from *Laminaria digitata* (Dectin-1 ligand, Sigma-Aldrich). The PRR agonists were used alone or in combination with 20 ng/mL mouse recombinant IFN-γ (Peprotech).

### A note on the detection limit of putative LPS contamination

The maximum amount of LPS that could potentially be present in the purified solutions of 100 µg/mL *I. obliquus* polysaccharides was determined to be 1.3 ng/mL LPS, as explained here. We have previously reported that the detection limit of LPS is 2 ng/µL when using GC-MS to detect the Lipid A part of the LPS molecule^28^. Any LPS amount lower than 2 ng/µL is not detected by GC-MS. After the isolation procedure was carried out for the polysaccharides, 5 mg polysaccharide was dissolved in 35 µL acetone solution, of which 1 µL was injected into the GC-MS instrument to detect possible LPS contamination. Thus, given the detection limit of 2 ng/µL LPS, the maximum amount of LPS that potentially could be present without being detected in our polysaccharide samples was < 70 ng per 5 mg polysaccharide (detection limit 2 ng/µL multiplied by 35 µL acetone polysaccharide solution = 70 ng LPS). In our in vitro and in vivo experiments, a concentration of 100 µg/mL polysaccharide was most often used. A LPS contamination of 70 ng in a 5 mg polysaccharide sample corresponds to a 1.4 ng LPS contamination in 100 µg polysaccharide sample. Because such a LPS contamination (1.4 ng LPS/μL) would have been detected by GC-MS, and because all our samples tested negative, we concluded that the maximum LPS concentration possible in our samples was 1.3 ng/mL.

### Quantification of NO

The Griess assay was used to measure NO production by activated macrophages, in the form of nitrite, as previously described with some modifications^28^. The BMDMs were seeded out in RPMI 1640 containing 10% FBS and 10% L929-derived conditioned medium, in a flat bottom 96-well plate (non-tissue culture treated, Costar) at cell density 6 ×104 cells to a final volume of 200 μL/well. After 24 h treatment with *I. obliquus* polysaccharides or other activation factors, cell culture media (100 μL) were collected from the wells and centrifuged (400 *g*, 2 min), and 50 μL supernatant was mixed with 50 μL of Griess reagent A (distilled H_2_O with 1% sulphanilamide [Sigma-Aldrich] and 5% phosphoric acid [Sigma-Aldrich]). The mixture was incubated in the dark for 10 min at room temperature, before 50 μL of Griess reagent B (0.1% *N*-(1-napthyl) ethylenediamine [Sigma-Aldrich] in distilled H_2_O) was added in order to convert NO into nitrite (NO_2_^−^), which was quantified colorimetrically at A_540_ using NaNO_2_ (1.56 – 100 μM) as a standard curve. Samples were set up in duplicates or triplicates depending on the experiment. For experiments using the LPS inhibitor polymyxin B (PMB, Polymyxin B sulfate salt, Sigma-Aldrich), the samples were mixed with 10 μg/mL PMB and incubated for 30 min at room temperature before being added to the cultivated cells. The experiments were carried out at least three times.

### In vitro growth inhibition assay

Wild-type (WT) and *Tlr4*^*−/−*^ mouse BMDMs were thawed and cultured for 3 days in non-tissue culture treated dishes (VWR) in RPMI 1640 medium containing 10% FBS and 10% L929-derived conditioned medium ( = complete medium). The macrophages were harvested by scraping, incubated for 2 h at 37 °C with mitomycin C (10 mg/mL, Sigma-Aldrich) to inhibit their proliferation, and then washed twice with PBS. Next, the macrophages were resuspended in complete medium and seeded out in triplicates in flat bottom 96-well plates (non-tissue culture treated, Costar) at three densities: 6 × 10^4^, 3 × 10^4^, and 3 × 10^3^ cells/well in a final volume of 200 μL/well. After 20 h, half of the medium was replaced with complete medium containing *I. obliquus* polysaccharides with or without IFN-γ. After incubation for 24 h, half of the cell supernatants (100 μL) were removed and used for quantification of nitrite (NO_2_^−^). Lewis lung carcinoma (LLC) cells (originally obtained from CLS Cell Lines Service, 3 × 10^3^ cells/well) were then added to the macrophages, resulting in varying ratios of effector to target cells: 20:1, 10:1 and 1:1. After 20 h of co-culture, ^3^H-thymidine (10 μL, 0.2 μCi/well, Hartmann Analytic) was added and the cells were harvested 24 h later after a freeze- and thaw cycle. The amount of radiolabeled DNA was measured on a 1450 MicroBeta Trilux Microplate Scintillation counter (Perkin Elmer). The experiments were carried out at least three times.

### In vivo growth inhibition assay

BMDMs were cultivated in non-tissue culture treated plates in RPMI 1640 containing 10% FBS (Biochrom, GmbH) and 10% L929-derived conditioned medium, and were stimulated for 24 h with AcF1 (100 μg/mL) + IFN-γ (20 ng/mL), AcF3 (100 μg/mL) + IFN-γ (20 ng/mL), IFN-γ (20 ng/mL) alone, Pam_3_CSK_4_ (100 ng/mL) + IFN-γ (20 ng/mL) (positive control) or left untreated (negative control). Lewis lung carcinoma (LLC) cells were grown under standard cell culture conditions in RPMI 1640 with 10% FBS, and harvested by trypzination. Macrophages were then mixed with LLC cells at a ratio of 15:1 in ice-cold PBS, and co-injected subcutaneously into 8-to-12-week-old, male and female C57BL/6NRj mice (7.5 × 10^5^ BMDMs + 5 × 10^4^ LLC cells per mouse). Tumor growth was monitored using a caliper every second or every third day until humane end points were reached (i.e. maximum tumor length = 15 mm). Formula for calculation of tumor size: Tumor volume (mm^3^) = [width^2^ × length] × 0.4 (ref. ^59^).

### Determination of iNOS mRNA levels by real-time quantitative PCR

Mouse BMDMs were seeded in 12-well plates (non-tissue culture treated, Sigma-Aldrich) at a density of 6 × 10^5^ cells/well in 1 mL RPMI 1640 medium supplemented with 10% FBS and 10% L929-derived conditioned medium. The cells were incubated for 2 h, before half of the medium was gently removed and replaced with 0.5 mL medium containing the indicated stimuli. After 24 h, cell culture media were removed and total RNA was extracted from the cells by using 300 µL/well of TRI Reagent (Merck) and Direct-zol RNA minipreps (Zymo Research) according to the manufacturer’s instructions. Next, mRNA concentrations were measured using Nanodrop One/One (Thermo Fisher), and 250 ng RNA of each sample was reverse-transcribed to cDNA using the Primescript RT kit (Takara Bio) according to the manufacturer’s instructions. Real-time quantitative PCR (qPCR) was performed with 50 ng of the obtained cDNA, using a Kapa SYBR fast qPCR kit (Kapa Biosystems) and 0.2 µM of mRNA specific primers for the mouse gene *Nos2* which encodes iNOS (forward primer: TTCACCCAGTTGTGCATC GACCTA, reverse primer: TCCATGGTCACCTCCAACACA AGA) and with primers for 18 s rRNA as the endogenous control (forward primer: CGCTTCCTTACCTGGTTGAT, reverse primer: GAGCGACCAAAGGAACCATA). Temperature and cycling conditions: 95 °C for 3 min; then in 40 cycles, 95 °C for 3 s, and 60 °C for 30 s. All samples were run in duplicates. Following melting curve analysis, the relative differences in iNOS mRNA levels were expressed using the - ΔCq values (Cq 18 s rRNA – Cq iNOS), where a more negative value means lower relative expression of iNOS mRNA compared to the housekeeping gene 18S rRNA. One unit increase in the negative ΔCq value corresponded to a doubling of iNOS mRNA. The experiment was carried out three times.

### Quantification of pro-inflammatory cytokines using Luminex technology

Mouse BMDMs, 2.5 × 10^5^ cells in 0.5 mL medium per well in 24-well plates (non-tissue culture treated Costar) were cultured in RPMI 1640 supplemented with 10% FBS (Biochrom) and 10% L929-derived conditioned medium. Before activation, BMDMs were treated with mitomycin C for 2 h. Human monocyte-derived macrophages, 1 × 10^5^ cells in 0.3 mL medium per well in 48-well plates (Costar) were cultured in RPMI 1640 supplemented with 10% FBS (Biowest). Mouse and human macrophages were stimulated for 24 h with polysaccharides ± IFN-γ. Cell culture media were collected and centrifuged (1000*g* for 15 min at 4 °C). Next, the supernatants were moved to new Eppendorf tubes and centrifuged again (1000*g* for 15 min at 4 °C) to remove cells and debris before storage at −80 °C until analysis. The concentrations of mouse and human IL-6 and TNF-α, and human IL-12p70, were determined by a multiplex Bio-Plex assay (Bio-Rad) according to the manufacturer’s instructions. Samples were analyzed in duplicates, using a Bio-Plex MAGPIX Multiplex Reader and Bio-Plex Manager 6.1 software (Bio-Rad Laboratories). The experiments were carried out two to three times.

### Reporter cell lines

HEK-Blue™ reporter cell lines (InvivoGen) transfected with human *TLR2*, human *Dectin1a*, human *TLR4/CD14/MD2* or non-transfected (null-1) were cultured and maintained using DMEM GlutaMAX™ containing 10% FBS (Sigma-Aldrich), Normocin (100 μg/mL) and HEK-Blue™ selection antibiotics. Experiments were carried out according to the manufacturer’s instructions. Briefly, *I. obliquus* polysaccharides (20 µL) at various concentrations were added to wells in 96-well plates (Costar). Then, the reporter cells were gently washed with warm PBS before suspended in HEK-Blue™ Secreted Embryonic Alkaline Phosphatase (SEAP) detection medium. Finally, 180 µL 5 × 10^4^ reporter cells were added per well containing polysaccharides. After incubation for 16 h (37 °C, 5% CO_2_), SEAP was detected colorimetrically at A_635_. The experiments were carried out at least three times.

### Statistics and reproducibility

Statistical analysis was conducted by using the GraphPad Prism 9.3.1 software (GraphPad). The results were analyzed using one-way ANOVA, followed by Dunn’s multiple comparison test, or calculated by a *t*-test followed by Bonferroni correction. The values were compared either across the data set or individually against the relevant controls depending on the experiment (stated specifically in figure legends). *P* < 0.05 was considered significant.

### Reporting summary

Further information on research design is available in the Nature Portfolio Reporting Summary linked to this article.

### Supplementary information

Supplementary Information

Description of Additional Supplementary Files

Supplementary Data

Reporting Summary

## Data Availability

The authors declare that the raw data supporting the findings of this study are available as a supplementary file (Supplementary Data 1).
