# Supplementary material for: Fungal polysaccharides from Inonotus obliquus are agonists for Toll-like receptors and induce macrophage anti-cancer activity
Source: Commun Biol. 2024 Feb 23;7:222. doi: 10.1038/s42003-024-05853-y (PMC10891174; doi:10.1038/s42003-024-05853-y)
Supplement: Supplementary file 1 — Supplementary Information [file 42003_2024_5853_MOESM1_ESM.pdf]

Fungal polysaccharides from *Inonotus obliquus* are agonists for Toll-like receptors and induce macrophage anti-cancer activity

Christian Winther Wold, Panagiotis F. Christopoulos, Maykel A. Arias, Deborah Elikplim Dzovor, Inger Øynebråten, Alexandre Corthay, and Kari Tvete Inngjerdingen

a

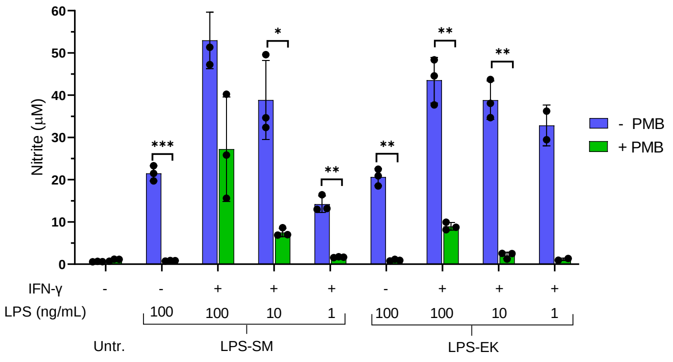

b

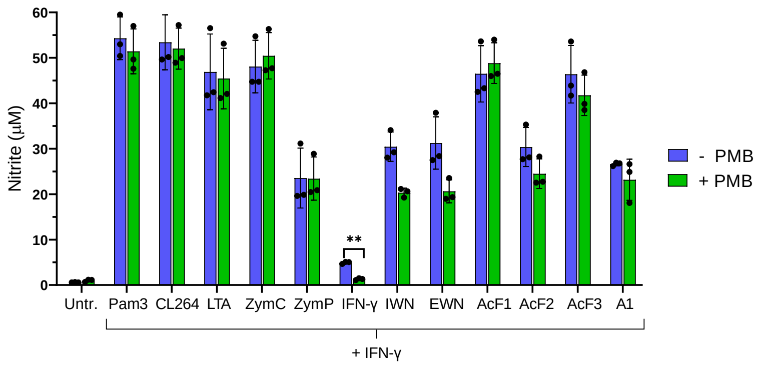

Supplementary Figure 1. Polymyxin B (PMB) treatment did not prevent *I. obliquus* polysaccharides from activating BMDMs, while LPS was strongly inhibited by the same treatment. PMB (final concentration 10 µg/mL) was incubated with the samples for 30 min at room temperature, before the samples were added to BMDMs. After incubation for 24 h, NO concentration was measured in the cell media using the Griess assay. **a**) LPS-induced activation of BMDMs was inhibited by PMB treatment. LPS from *S. minnesota* (LPS-SM) and *E. coli* (LPS-EK) were used at 1, 10 and 100 ng/mL in combination with IFN-γ (20 ng/mL), or at 100 ng/mL alone. Three independent experiments were performed, and average values ± SD are shown. **b**) Polysaccharides from *I. obliquus* or various PRR ligands were able to activate BMDMs in combination with IFN-γ, even in the presence of PMB. Polysaccharides from *I. obliquus* were used at 100 µg/mL. Controls: Pam_3CSK_4 (Pam_3, 100 ng/mL, a TLR1/2 agonist); CL264 (1 µg/mL, a TLR7 agonist); LTA (100 µg/mL, a TLR2 agonist); zymosan crude (ZymC, 100 µg/mL, a TLR2/Dectin-1 agonist); zymosan purified (ZymP, 100 µg/mL, a Dectin-1 agonist); IFN-γ (20 ng/mL). (**a)** and (**b)** are from the same experiments; the graphs are shown separately to highlight how PMB affects LPS in a dose-dependent manner. In **(a)** and **(b)** statistical significance between + and – PMB conditions for each individual sample and sample concentration was calculated by a t-test followed by Bonferroni correction. *** = $p < 0.001$, ** = $p < 0.01$, * = $p < 0.05$.
