# Supplementary material for: Fungal polysaccharides from Inonotus obliquus are agonists for Toll-like receptors and induce macrophage anti-cancer activity
Source: Commun Biol. 2024 Feb 23;7:222. doi: 10.1038/s42003-024-05853-y (PMC10891174; doi:10.1038/s42003-024-05853-y)
Supplement: Supplementary file 4 — Reporting Summary [file 42003_2024_5853_MOESM4_ESM.pdf]

nature portfolio

Corresponding author(s): Christian Winther Wold, Alexandre Corthay, and Kari Tvete Inngjerdingen

Last updated by author(s): 2024/01/18

Reporting Summary

Nature Portfolio wishes to improve the reproducibility of the work that we publish. This form provides structure for consistency and transparency in reporting. For further information on Nature Portfolio policies, see our Editorial Policies and the Editorial Policy Checklist.

Statistics

For all statistical analyses, confirm that the following items are present in the figure legend, table legend, main text, or Methods section.

n/a	Confirmed	
☐	☒	The exact sample size (*n*) for each experimental group/condition, given as a discrete number and unit of measurement
☐	☒	A statement on whether measurements were taken from distinct samples or whether the same sample was measured repeatedly
☐	☒	The statistical test(s) used AND whether they are one- or two-sided<br>*Only common tests should be described solely by name; describe more complex techniques in the Methods section.*
☒	☐	A description of all covariates tested
☐	☒	A description of any assumptions or corrections, such as tests of normality and adjustment for multiple comparisons
☐	☒	A full description of the statistical parameters including central tendency (e.g. means) or other basic estimates (e.g. regression coefficient) AND variation (e.g. standard deviation) or associated estimates of uncertainty (e.g. confidence intervals)
☐	☒	For null hypothesis testing, the test statistic (e.g. *F*, *t*, *r*) with confidence intervals, effect sizes, degrees of freedom and *P* value noted<br>*Give P values as exact values whenever suitable.*
☒	☐	For Bayesian analysis, information on the choice of priors and Markov chain Monte Carlo settings
☒	☐	For hierarchical and complex designs, identification of the appropriate level for tests and full reporting of outcomes
☒	☐	Estimates of effect sizes (e.g. Cohen's *d*, Pearson's *r*), indicating how they were calculated

Our web collection on statistics for biologists contains articles on many of the points above.

Software and code

Policy information about availability of computer code

Data collection	No software was used.
Data analysis	Statistical analysis was conducted by using the GraphPad Prism 9.3.1 software (GraphPad). The results were analyzed using one-way ANOVA, followed by Dunn's multiple comparison test, or by a t-test followed by Bonferroni correction. The values were compared either across the data set or individually against the relevant controls depending on the experiment (stated specifically below each experiment figure). P<0.05 was considered significant.

For manuscripts utilizing custom algorithms or software that are central to the research but not yet described in published literature, software must be made available to editors and reviewers. We strongly encourage code deposition in a community repository (e.g. GitHub). See the Nature Portfolio guidelines for submitting code & software for further information.

Data

Policy information about availability of data

All manuscripts must include a data availability statement. This statement should provide the following information, where applicable:

- Accession codes, unique identifiers, or web links for publicly available datasets
- A description of any restrictions on data availability
- For clinical datasets or third party data, please ensure that the statement adheres to our policy

The numerical source data for graphs in the manuscript are are available as a supplementary file (Supplementary data 1).

Human research participants

Policy information about studies involving human research participants and Sex and Gender in Research.

Reporting on sex and gender	n/a
Population characteristics	Anonymized healthy blood donors
Recruitment	Blood (buffy coats) from anonymised healthy donors was obtained from the Blood bank of Oslo University Hospital to generate monocyte-derived macrophages for in vitro studies.
Ethics oversight	The use of blood (buffy coats) from anonymised healthy donors was approved by the Norwegian Regional Committee for Medical and Health Research Ethics (REK no. 2019/113).

Note that full information on the approval of the study protocol must also be provided in the manuscript.

Field-specific reporting

Please select the one below that is the best fit for your research. If you are not sure, read the appropriate sections before making your selection.

☒ Life sciences ☐ Behavioural & social sciences ☐ Ecological, evolutionary & environmental sciences

For a reference copy of the document with all sections, see nature.com/documents/nr-reporting-summary-flat.pdf

Life sciences study design

All studies must disclose on these points even when the disclosure is negative.

Sample size	Sample size was determined based on previous work by the authors who have many years of experience in the field. We aimed for sufficiently large sample size to be able to identify statistically significant differences between the groups, while keeping the experiments technically feasible by not having too large groups.
Data exclusions	n/a
Replication	Independent experiments were performed to verify reproducibility, as stated in the figure legends.
Randomization	For the mouse experiments, mice were randomly allocated to experimental groups.
Blinding	Tumor growth in mice was measured in a blinded fashion.

Reporting for specific materials, systems and methods

We require information from authors about some types of materials, experimental systems and methods used in many studies. Here, indicate whether each material, system or method listed is relevant to your study. If you are not sure if a list item applies to your research, read the appropriate section before selecting a response.

Materials & experimental systems

n/a	Involved in the study	
☒	☐	Antibodies
☐	☒	Eukaryotic cell lines
☒	☐	Palaeontology and archaeology
☐	☒	Animals and other organisms
☒	☐	Clinical data
☒	☐	Dual use research of concern

Methods

n/a	Involved in the study	
☒	☐	ChIP-seq
☒	☐	Flow cytometry
☒	☐	MRI-based neuroimaging

Eukaryotic cell lines

Policy information about cell lines and Sex and Gender in Research

Cell line source(s)	HEK-Blue™ reporter cell lines transfected with human TLR2, human Dectin1a, human TLR4/CD14/MD2 or non-transfected (null-1) were purchased from Invivogen.

Authentication	The cell lines were authenticated using specific agonist for the transfected receptors.
Mycoplasma contamination	All cell lines tested negative for mycoplasma contamination.
Commonly misidentified lines (See ICLAC register)	n/a

Animals and other research organisms

Policy information about studies involving animals; ARRIVE guidelines recommended for reporting animal research, and Sex and Gender in Research

Laboratory animals	C57BL/6NRj mice were purchased from Janvier Labs (Le Genest-Saint-Isle, France) and bred at the Department of Comparative Medicine, Oslo University Hospital, Rikshospitalet (Oslo, Norway) in specific pathogen free (SPF) conditions. C57BL/6NRj mice deficient in TLR4 (Tlr4-/-) [30] were bred at the University of Zaragoza, Zaragoza, Spain.
Wild animals	n/a
Reporting on sex	Mice of both sexes were used.
Field-collected samples	n/a
Ethics oversight	The study was approved by the Norwegian Food Safety Authority (approval number 20/102031), and all the experiments were performed in accordance with the national regulations and the EU directive 2010/63/EU.

Note that full information on the approval of the study protocol must also be provided in the manuscript.
